# Comprehensive analysis of the transcriptome‐wide m^6^A methylome in invasive malignant pleomorphic adenoma

**DOI:** 10.1186/s12935-021-01839-6

**Published:** 2021-03-02

**Authors:** Zhenyuan Han, Biao Yang, Qin Wang, Yuhua Hu, Yuqiong Wu, Zhen Tian

**Affiliations:** 1 Department of Oral Pathology, Shanghai Ninth People’s Hospital, Shanghai Jiao Tong University School of Medicine, Shanghai, 200011 China; 2 National Clinical Research Center for Oral Diseases, Shanghai, 200011 China; 3 Department of Neurosurgery, Huashan Hospital of Fudan University, Shanghai, 200040 China; 4 Clinical Translational Research Center, Shanghai Pulmonary Hospital, School of Life Sciences and Technology, Tongji University, Shanghai, 200092 China; 5 Department of Prosthodontics, Shanghai Ninth People’s Hospital, Shanghai Jiao Tong University School of Medicine, Shanghai, 200011 China

**Keywords:** Malignant pleomorphic adenoma, N^6^-Methyladenosine, MeRIP-seq

## Abstract

**Background:**

Invasive malignant pleomorphic adenoma (IMPA) is a highly invasive parotid gland tumor and lacks effective therapy. N6-Methyladenosine (m^6^A) is the most prevalent post-transcriptional modification of mRNAs in eukaryotes and plays an important role in the pathogenesis of multiple tumors. However, the significance of m^6^A-modified mRNAs in IMPA has not been elucidated to date. Hence, in this study, we attempted to profile the effect of IMPA in terms of m^6^A methylation in mRNA.

**Methods:**

Methylated RNA immunoprecipitation with next-generation sequencing (MeRIP-seq) and RNA sequencing (RNA-seq) were utilized to acquire the first transcriptome-wide profiling of the m^6^A methylome map in IMPA followed by bioinformatics analysis.

**Results:**

In this study, we obtained m^6^A methylation maps of IMPA samples and normal adjacent tissues through MeRIP-seq. In total, 25,490 m^6^A peaks associated with 13,735 genes were detected in the IMPA group, whereas 33,930 m^6^A peaks associated with 18,063 genes were detected in the control group. Peaks were primarily enriched within coding regions and near stop codons with AAACC and GGAC motifs. Moreover, functional enrichment analysis demonstrated that m^6^A-containing genes were significantly enriched in cancer and metabolism relevant pathways. Furthermore, we identified a relationship between the m^6^A methylome and the RNA transcriptome, indicating a mechanism by which m^6^A modulates gene expression.

**Conclusions:**

Our study is the first to provide comprehensive and transcriptome-wide profiles to determine the potential roles played by m^6^A methylation in IMPA. These results may open new avenues for in-depth research elucidating the m^6^A topology of IMPA and the molecular mechanisms governing the formation and progression of IMPA.

## Introduction

Pleomorphic adenoma (PA), one of the most common types of salivary neoplasms, typically occurs in the superficial lobe of the parotid gland and accounts for 60–70% of all parotid tumors [1]. Although initially benign, long-standing PA has the potential to transform into malignant pleomorphic adenoma (MPA) and subsequently infiltrate surrounding facial structures, which occurs in 6% of patients [2, 3]. MPA can be further classified into three subtypes according to the depth of tumor infiltration beyond the capsule of the previous PA, namely, non-invasive, micro-invasive, and invasive malignant pleomorphic adenoma (IMPA) [4]. For IMPA, it is well accepted that surgical excision is the first-line treatment modality, but its high incidence of local recurrence and distant metastasis after surgery is a thorny problem that remains unsolved to date [5]. Besides, traditional molecular-targeted drugs against estrogen receptors (ER) or human epidermal growth factor receptor 2 (HER2) are of limited efficiency in treating IMPA, since a variety of patients are negative for both ER and HER2. To circumvent these obstacles, further research is necessary to identify a new molecular basis of IMPA.

Reversible RNA modifications, including N1-Methyladenosine (m^1^A), 5-Methylcytosine (m^5^C), and N6-Methyladenosine (m^6^A), have garnered substantial attention from oncologists in recent years [6]. Among these modifications, m^6^A is recognized as the most prevalent internal modification of poly-adenylated mRNAs and long non-coding RNAs in eukaryotic cells. Since it was first identified by Wei et al. in the 1970s, m^6^A has been deciphered to modulate various biological processes, such as adipogenesis, circadian rhythm and embryonic stem cell self-renewal and differentiation [7]. Mechanistically, the m^6^A RNA modification is enzymatically induced by m^6^A “writers”, a group of methyltransferases. METTL3, METTL14 and WTAP form the heterodimer core complex and mediate m^6^A deposition on mammalian nuclear RNAs [8, 9]. Next, m^6^A “readers” selectively recognize m^6^A-modified RNAs in the cytoplasm and exert regulatory functions by affecting multiple aspects of RNA metabolism, such as RNA stability, translation, transport and splicing. YTHDF1, YTHDC2, IGF2BP1/2/3, and HNRNPC are the primary “readers” in eukaryotic cells [10–12]. Notably, mammalian m^6^A modification is dynamic and reversible. Various demethylases, collectively known as m^6^A “erasers”, have been identified to participate in the process by removing N^6^-Methyladenosine from RNAs in the nucleus; these demethylases, include FTO and ALKBH5 [13].

Recently, m^6^A has been demonstrated to play a significant role in the development and progression of many tumors. Chen et al. reported the highly diverse m^6^A modification patterns between clear cell renal cell carcinoma (ccRCC) and tumor-adjacent normal tissue. Newly characterized m^6^A peaks in ccRCC have been proven to modulate gene expression and cancer-related pathways [14]. Similarly, thousands of dysregulated m^6^A peaks, which can be detected in colorectal cancer (CRC) samples, are associated with the prognosis of CRC patients [15]. In addition, the well-studied m^6^A “writer” METTL3 has been positively associated with leukemogenesis of acute myeloid leukemia and represents a therapeutic target in the disease [16]. Depletion of METTL3 in human myeloid leukemia cell lines induces differentiation and apoptosis and even delays leukemia progression in vivo [16]. Furthermore, the m^6^A levels of mRNAs regulated by METTL3 may also affect leukemogenesis [16]. Mutations of the two m^6^A “erasers” FTO and ALKBH5, as demonstrated by Cui et al., can facilitate malignant transformation of glioblastomas by promoting glioblastoma stem cell growth, self-renewal and tumor progression [17]. Another study conducted by Zhao et al. confirmed the oncogenic role of the m^6^A “reader” protein YTHDF1 in hepatocellular carcinoma, but further research is necessary [18].

Given that m^6^A modification is a dynamic and reversible process, reversing abnormal m^6^A patterns in tumors represents a promising strategy for targeted therapy. To date, pharmacologically targeting m^6^A regulators has been proven to be efficacious in both in vitro and in vivo studies [19]. Meclofenamic acid 2 (MA2) is an FDA-approved NSAID, that binds to the surface area of the FTO active site and selectively inhibits FTO. Treating glioblastoma stem cells with MA2 causes an increase in m^6^A levels, leading to a significant reduction in cell growth and self-renewal [17, 20]. CHTB, N-CDPCB and R-2HG are other FTO inhibitors with potential for clinical application [21]. No inhibitors of other m^6^A regulators are currently available, but targeting aberrant m^6^A modification in tumors is a feasible approach and warrants further research.

Limited knowledge is available regarding RNA m^6^A modification profiles in IMPA. Given the indispensable role of RNA m^6^A modification in the onset and progression of a wide variety of tumors, it is reasonable to surmise that the dysregulation of m^6^A modification might be associated with IMPA. To investigate the relationship between m^6^A and IMPA, we delineated the first-ever m^6^A transcriptome-wide landscape of IMPA. The function of the new layer of differentially methylated peaks in IMPA was also determined in our work.

## Materials and methods

### Specimens collections and ethics statement

Five pairs of IMPA tumors and the corresponding normal tissues were acquired at the time of surgery from Shanghai Ninth People’s Hospital, Shanghai Jiao Tong University School of Medicine between 5 Jan 2019 and 5 Oct 2019. After surgery, the candidate tissues were immediately separated into RNase-free centrifuge tubes, snap-frozen in liquid nitrogen and stored at − 80 °C until RNA isolation. Furthermore, all sample sections (4 µm) were stained with hematoxylin and eosin (H&E) and histologically reviewed by two pathologists. This study was approved by the Ethics Committee of Shanghai Ninth People’s Hospital.

### MeRIP-seq and RNA-seq

Total RNA from each sample was isolated and purified using TRIzol reagent (Invitrogen, Carlsbad, CA, USA) following the instructions of the manufacturer. Next, the amount and quality of the RNA from each sample were quantified using a NanoDrop ND-1000 (NanoDrop, Wilmington, DE, USA) instrument. RNA integrity was evaluated by a Bioanalyzer 2100 (Agilent, CA, USA) based on the threshold of RIN number > 7.0 and confirmed by electrophoresis with a denaturing agarose gel. Next, poly (A) RNAs were purified from 50 µg of total RNA by applying Dynabeads Oligo (dT) 25-61005 (Thermo Fisher, CA, USA) using two rounds of purification. Then, by using Magnesium RNA Fragmentation Module (NEB, cat.e6150, USA) at 86 ℃ for 7 min, the poly(A) RNA was fragmented into small pieces. After that step, the fragmented RNAs were incubated for 2 h at 4 ℃ in the presence of an m^6^A-specific antibody (No. 202003, Synaptic Systems, Germany) in IP buffer (50 mM Tris-HCl, 750 mM NaCl and 0.5 % Igepal CA-630). A portion of the initial fragmented RNA was used as the input RNA-seq library for MeRIP-seq. Next, the IP RNA was reverse-transcribed to synthesize cDNA by SuperScript™ II Reverse Transcriptase (Invitrogen, cat. 1896649 USA), which were subsequently used to create U-labeled second-stranded DNAs with *E. coli* DNA polymerase I (NEB, cat.m0209, USA), RNase H (NEB, cat.m0297, USA) and dUTP Solution (Thermo Fisher, cat.R0133, USA). Furthermore, an A-base was added to the blunt ends of each strand for the purpose of ligation to the indexed adapters. Each adapter contains a T-base overhang for ligating the adapter to the specific A-tailed fragmented DNA. Single- or dual-index adapters were ligated to the piece of fragments, and size selection and purity were performed with protein-A beads. After heat-labile UDG enzyme (NEB, cat.m0280, USA) treatment of the U-labeled second-stranded DNAs, the ligated products were amplified via PCR technology under the following conditions: (1) initial denaturation at 95 ℃ for 3 min; (2) 8 cycles of denaturation at 98 ℃ for 15 s, annealing at 60 ℃ for 15 s, and extension at 72 ℃ for 30 s; (3) and then final extension at 72 ℃ for 5 min. The average insert size for the final cDNA library was 300 ± 50 bp. Finally, both the input tissues without immunoprecipitation and the IP tissues were subjected to the 2 × 150-bp paired-end sequencing (PE150) on an Illumina NovaSeq™ 6000 (LC-Bio Technology Co., Ltd., Hangzhou, China) following the manufacturer’s instructions.

### Bioinformatics analysis process

After the paired-end reads were obtained from Illumina NovaSeq™ 6000, the clean data were harvested using the Fastq (https://github.com/OpenGene/fastp) package to perform quality control, including adapter-trimming, and removal of low-quality reads in fastq format. After that step, we utilized HISAT2 (http://daehwankimlab.github.io/hisat2) software to align the clean reads to the reference genome (version: hg38). Next, the mapped reads of IP and input libraries were provided for the R package exomePeak (https://bioconductor.org/packages/exomePeak), which served as m^6^A peak calling in bed or bigwig format that can be adapted for visualization via the Integrative Genomics Viewer (IGV) software (http://www.igv.org). Differentially methylated peaks were also identified via exomePeak according to the criteria |log_2_Fold Change (FC) | > 1 and p-value < 0.05. Furthermore, the MEME (http://meme-suite.org) and HOMER (http://homer.ucsd.edu/homer/motif) packages were used for de novo and known motif finding followed by localization of the motif concerning peak summit. Peaks were annotated by the intersection with gene architecture utilizing the R package ChIPseeker (https://bioconductor.org/packages/ChIPseeker). Next, StringTie (https://ccb.jhu.edu/software/stringtie) was applied to perform expression level for all mRNAs from input libraries by calculating FPKM (total exon fragments/mapped reads (millions) × exon length (kB)). Finally, the differentially expressed genes were identified with |log_2_FC| > 1 and p-value < 0.05 by the edgeR (https://bioconductor.org/packages/edgeR) package in R.

### Public databases and analysis

The Database for Annotation, Visualization and Integrated Discovery (DAVID) database (https://david.ncifcrf.gov/) was used in Gene Oncology (GO) functional enrichment analysis and Kyoto Encyclopedia of Genes and Genomes (KEGG) pathway analysis for genes associated with m^6^A-modified peaks. Regarding the functional enrichment, the GO covered three domains: biological process (BP), cellular component (CC), and molecular function (MF). The RMBase v2.0 (http://rna.sysu.edu.cn/) database was applied to predict the m^6^A modification site in BACE2. All the functional terms were regarded as significant as the threshold of p-value < 0.05.

## Results

### Overall features of m^6^A methylation in IMPA

**Fig. 1:**
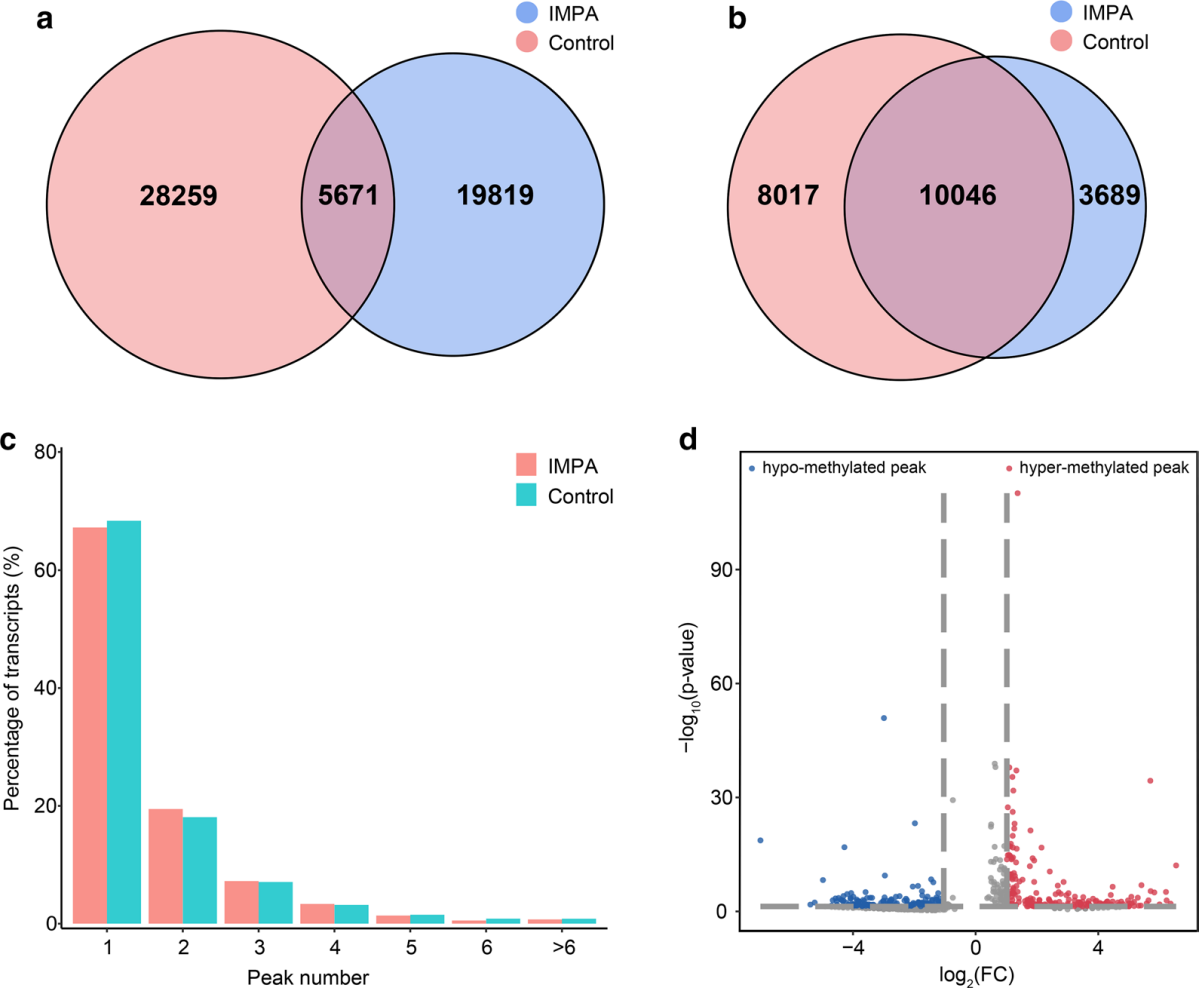
Overview of m^6^A methylation modification in IMPA and adjacent normal control tissues. **a**, **b** Venn diagrams showing 5671 overlaps of m^6^A peaks (**a**) and 10,046 overlaps of m^6^A-modified genes (**b**) between IMPA and normal control tissues, respectively. **c** Number of peaks per transcript in IMPA and normal control tissues. **d** Identification of 183 hyper-methylated and 150 hypo-methylated m^6^A peaks in five pairs of IMPA and corresponding normal control, which presented a significant alteration, respectively (|log_2_ FC | > 1, p < 0.05)

After the raw data were filtered and corrected, more than 35,000,000 high-quality reads acquired from each sample were mapped to the hg38 genome (Table 1). Of these high-value data, 5671 m^6^A methylated peaks were detected based on the pairwise comparison of read distribution between MeRIP-seq and RNA-seq samples in both tumor and corresponding normal tissues (Fig. 1a). Furthermore, 25,490 m^6^A modified peaks were identified in the IMPA group, which were related to 13,735 genes. Similarly, 33,930 m^6^A modified peaks were identified among 18,063 transcripts of genes in the control group (Fig. 1a, b). The differences and overlap in m^6^A modified genes were demonstrated in Fig. 1b (Additional file 1: Table S1). Among these, we discovered that only 10,046 m^6^A modified genes were generally expressed in both groups. The top 20 differentially modified peaks are listed in Table 2. Taken together, these results indicate an apparent discrepancy in global m^6^A modification patterns between IMPA and corresponding normal tissues.

**Table 1:** Summary of sequencing data and reads alignment statistics from MeRIP-seq and RNA-seq of IMPA tumors and corresponding normal tissues

Sample_ID	Raw_Reads	Raw_Bases	Valid_Reads	Valid_Bases	Valid%	Q30%	GC%
N_1_input	37,981,486	5.70G	36,680,474	4.25G	74.54	92.56	60.10
N_1_IP	42,442,892	6.37G	40,978,956	4.71G	73.99	92.75	56.38
N_2_input	46,504,552	6.98G	44,899,032	5.11G	73.23	93.46	61.22
N_2_IP	43,737,914	6.56G	41,800,672	5.29G	80.63	92.84	42.40
N_3_input	52,307,560	7.85G	51,282,842	7.01G	89.28	93.05	49.66
N_3_IP	54,502,114	8.18G	52,615,686	7.31G	89.45	92.87	51.81
N_4_input	52,283,724	7.84G	50,587,688	6.84G	87.25	92.23	51.54
N_4_IP	53,657,420	8.05G	52,397,276	7.26G	90.15	91.86	53.12
N_5_input	47,355,842	7.10G	46,405,300	6.36G	89.52	93.67	48.27
N_5_IP	45,001,614	6.75G	44,176,592	6.17G	91.38	93.67	50.94
T_1_input	37,010,304	5.55G	35,760,348	4.32G	77.79	93.35	51.39
T_1_IP	42,144,148	6.32G	41,469,572	5.06G	80.01	94.84	48.96
T_2_input	38,337,450	5.75G	37,256,256	4.56G	79.30	93.73	53.78
T_2_IP	43,666,036	6.55G	42,549,088	5.26G	80.26	94.29	50.59
T_3_input	47,030,148	7.05G	45,993,690	6.31G	89.41	93.59	49.06
T_3_IP	48,856,372	7.33G	47,336,610	6.61G	90.18	93.44	50.77
T_4_input	47,752,230	7.16G	46,666,654	6.37G	88.96	93.78	48.72
T_4_IP	50,768,676	7.62G	49,769,428	6.96G	91.33	93.60	50.39
T_5_input	52,662,714	7.90G	51,546,390	7.07G	89.55	93.77	49.49
T_5_IP	50,883,836	7.63G	48,867,432	6.83G	89.43	93.66	51.21

N represents the IMPA normal tissues, while T represents the IMPA tumor tissues. Q30 % reflects the percentage of bases with a quality value ≥ 30 (sequencing error rate less than 0.001)

**Table 2:** The top 20 differently methylated m6A peaks (IMPA tumor/normal)

Gene name	Gene ID	log_2_ Fold Change	Regulation	Chromosome	Peak_start	Peak_end	p-value
BACE2	ENSG00000182240	6.54	Up	Chr 21	41,186,548	41,186,698	0.00
ALOX12B	ENSG00000179477	6.36	Up	Chr 17	8,079,810	8,080,277	0.01
TNXA	ENSG00000248290	6.23	Up	Chr 6	32,010,489	32,010,726	0.00
DOCK7	ENSG00000116641	6.21	Up	Chr 1	62,641,179	62,641,409	0.00
HIST2H3DP1	ENSG00000213244	6	Up	Chr 1	121,118,184	121,128,206	0.01
FP565260	ENSG00000280433	5.83	Up	Chr 21	5,158,219	5,159,167	0.00
CBSL	ENSG00000274276	5.71	Up	Chr 21	6,452,125	6,452,215	0.00
ANKRD36B	ENSG00000196912	5.70	Up	Chr 2	97,507,998	97,511,336	0.00
FOXD4L4	ENSG00000184659	5.64	Up	Chr 9	65,737,149	65,737,328	0.00
AL365475	ENSG00000261208	5.58	Up	Chr 14	52,287,157	52,287,277	0.01
FAM156A	ENSG00000268350	− 7.02	Down	Chr X	52,956,902	52,957,560	0.00
HELLPAR	ENSG00000281344	− 5.39	Down	Chr 12	102,300,840	102,301,020	0.02
LINC02006	ENSG00000238755	− 5.25	Down	Chr 3	153,385,144	153,385,323	0.00
CR381653	ENSG00000279208	− 4.98	Down	Chr 21	9,375,718	9,376,586	0.00
AL590440	ENSG00000284601	− 4.70	Down	Chr 1	54,975,109	54,975,527	0.03
MYH2	ENSG00000125414	− 4.66	Down	Chr 17	10,529,982	10,531,659	0.00
FAM27C	ENSG00000231527	− 4.57	Down	Chr 9	61,855,062	61,855,122	0.00
HNRNPA0	ENSG00000177733	− 4.54	Down	Chr 5	137,748,140	137,748,349	0.00
LINC01894	ENSG00000264345	− 4.52	Down	Chr 18	24,987,534	24,987,682	0.02
AL356585	ENSG00000279924	− 4.47	Down	Chr 13	18,177,806	18,178,285	0.00

To characterize the variation in the number of m^6^A-modified peaks in the transcript, we analyzed MeRIP-seq results and observed that approximately 67% of all modified transcripts had one m^6^A peak. To go one step further, a relatively large number of genes (93.7%) harbored 1 to 3 m^6^A-modified sites (Fig. 1c, Additional file 2: Table S2). Next, the abundance of m^6^A peaks between IMPA and adjacent normal control samples was analyzed with the norm of |log_2_FC| > 1 and p < 0.05. A total of 183 hyper-methylated peaks and 150 hypo-methylated peaks were depicted in IMPA samples compared with the control groups (Fig. 1d, Additional file 3: Table S3).

### Topological patterns of IMPA methylation

**Fig. 2:**
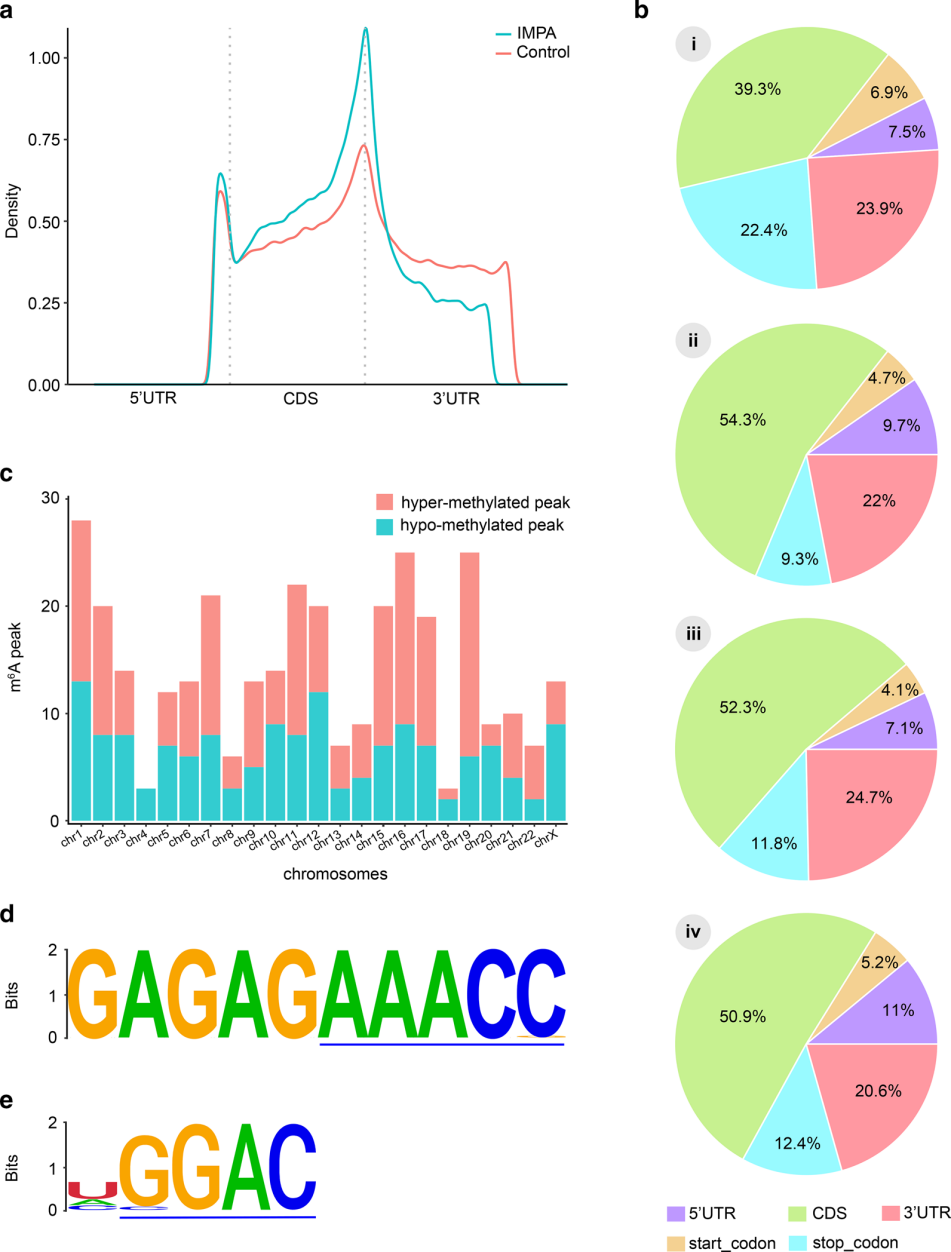
Distribution and topology pattern of m^6^A peaks along transcripts and chromatin in IMPA and the corresponding normal tissues. **a** Difference in the density of m^6^A peaks along the transcript. Each transcript was divided into three parts: 5’-UTR, CDS, and 3’-UTR. **b** Pie charts showing the distributions of m^6^A peaks in the whole transcriptome of IMPAs and controls (i), m^6^A peaks in IMPAs (ii), m^6^A peaks in controls (iii), and differentially methylated m^6^A peaks between IMPAs and controls (iv). **c** The distribution of differentially methylated m^6^A peaks with significance along the chromosome of IMPA. **d**,** e** Sequencing logo displaying AAACC and GGAC conserved sequence motifs for the m^6^A peak regions, with p-values of 1*e^− 301^ and 1*e^− 203^, respectively

The topological patterns of m^6^A modification in IMPA was investigated, the results presented in Fig. 2a demonstrate that the distribution of m^6^A peaks was principally enriched in coding sequence (CDS) regions close to the stop codon regardless of the IMPA tumor or normal tissue origin. For a more precise evaluation of m^6^A peaks, both m^6^A-modified peaks in the general and unique groups were analyzed according to the transcript’s location. Overall, the m^6^A sites were primarily deposited in the CDS, 3′-UTR, and stop codon (Fig. 2b i). However, the IMPA groups exhibited a distinct pattern from the control group with a relative increase in m^6^A enrichment harbored in the vicinity of the CDS region but less m^6^A enrichment near the stop codon (Fig. 2b ii, 2b iii). Besides, the differentially methylated m^6^A sites in the two groups most often appeared in the CDS (50.9%), followed by the 3′-UTR (20.6 %) (Fig. 2b iv). The altered m^6^A peaks were further observed to be mapped to all human chromosomes. The top three chromosomes sharing the most hyper-methylated and hypo-methylated m^6^A peaks within mRNA were chromosomes 1, 16, and 19, and hyper-methylated m^6^A peaks were observed in all chromosome but chromosome 4 (Fig. 2c, Additional file 4: Table S4). Additionally, the HOMER package was applied to search for consensus motifs deposited in the region surrounding the m^6^A peak and to identify the classical AAACH and GGAC motif structures (Fig. 2d, e), which was in accord with the previous findings. The identification of a strong consensus strengthens the authenticity of the identified m^6^A peaks and suggest the existence of a dominant methylation mechanism.

### m^6^A-containing genes are involved in important biological processes and pathways

**Fig. 3:**
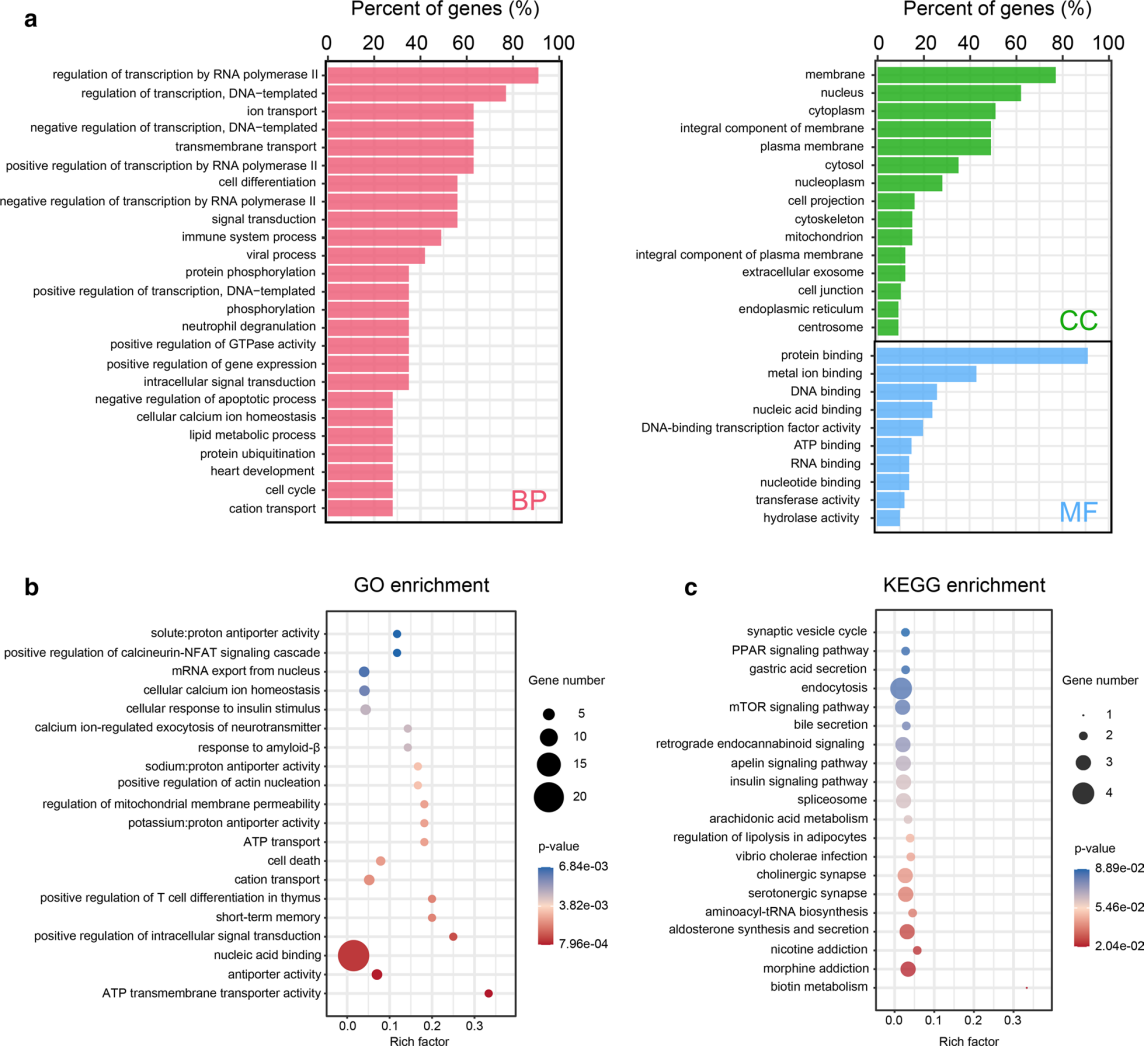
GO function and KEGG pathway enrichment of hyper-methylated m^6^A genes. **a** Major enrichment and meaningful GO terms of hyper-methylated m^6^A genes in IMPA. **b** The top twenty significant GO enrichment terms. **c** The top twenty significant KEGG pathways

In this study, transcriptome features of the abnormal m^6^A-modified genes in IMPA were determined by MeRIP-seq. To investigate the biological significance of m^6^A-containing genes in IMPA, the hyper-methylated transcripts were subjected the GO and KEGG analyses. With GO analysis, genes were divided into three functional domains: biological process (BP), cellular component (CC), and molecular function (MF). Figure 3a (Additional file 5: Table S5) lists the top 25 BP terms, top 15 CC terms, and top 10 MF terms of the hyper-methylated genes which indicated that those genes were significantly enriched in ATP transmembrane transporter activity, antiporter activity, and nucleic acid binding (Fig. 3b, Additional file 6: Table S6). With respected to the KEGG pathways of genes with up-methylated m^6^A peaks, we found that the hyper-methylated mRNAs were enriched in biotin metabolism, morphine addiction, nicotine addiction, aldosterone synthesis and secretion, the PPAR signaling pathway, and the mTOR signaling pathway (Fig. 3c, Additional file 7: Table S7). To sum up, the enrichment results may serve to increase the scientific interest in RNA m^6^A modification in IMPA.

### Overview of transcriptome profiles and conjoint analysis of m^6^A methylation and gene expression

**Fig. 4:**
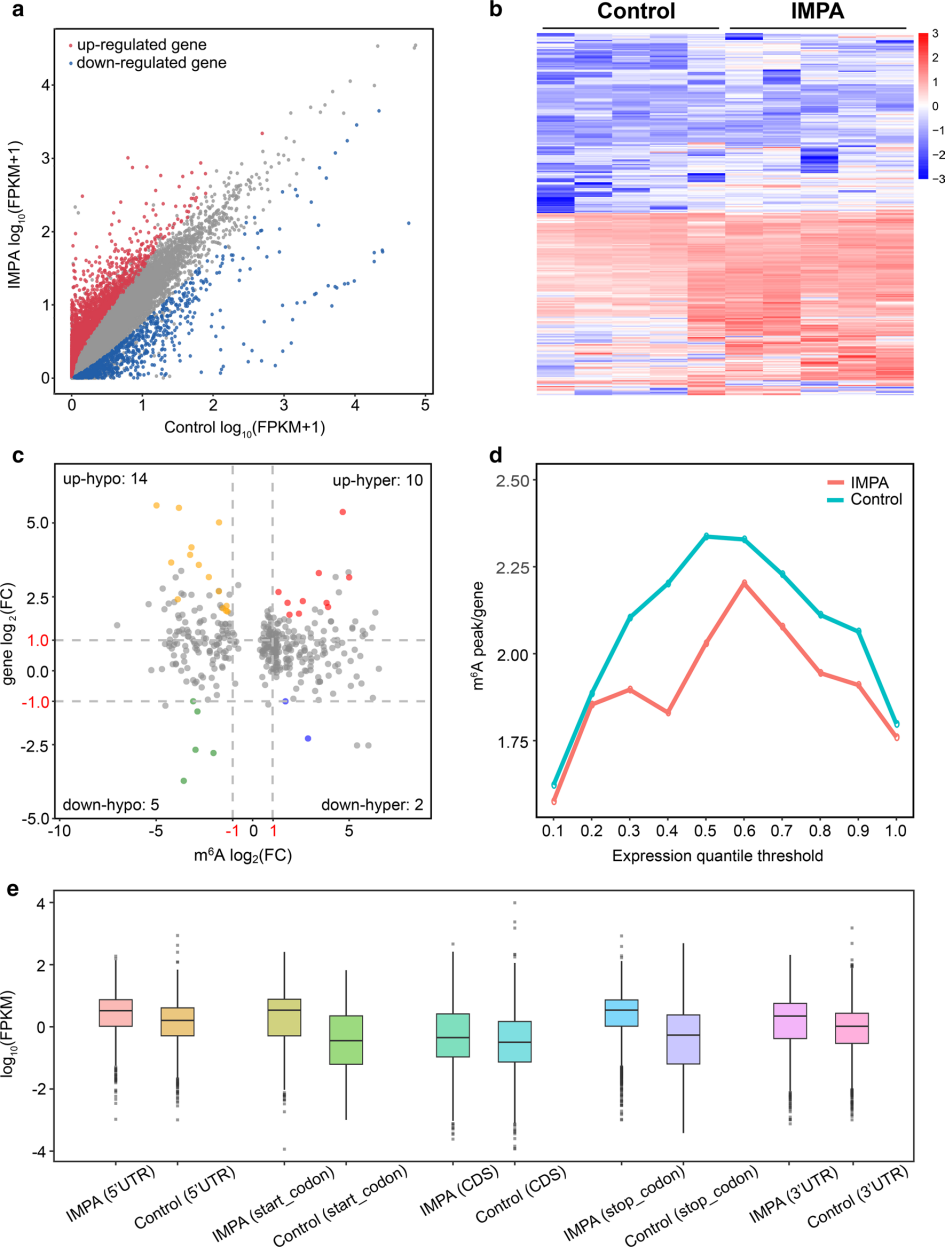
Comprehensive analysis of the m^6^A methylome and RNA transcriptome in IMPA. **a**,** b** Scatter plot and hierarchical clustering presenting the differentially expressed genes in IMPA. **c** Four quadrant graph exhibiting the distribution of mRNAs with a significant alteration in both m^6^A and mRNA levels between IMPA and adjacent normal tissues (|log_2_ FC| > 1, p < 0.05). **d** Estimation of m^6^A peaks density in IMPA. **e** Expression levels of genes containing m^6^A-specific peaks in different five regions of transcript between IMPA and normal tissues

To detect the transcriptome profiles of IMPA samples versus the corresponding normal tissues from five pairs of IMPA patients, we employed RNA-seq (MeRIP-seq input library) to quantify the differential gene expression in the 2 groups. Compared with normal control samples, 3028 genes were differentially expressed in IMPA (|log_2_FC| > 1, and p < 0.05), including 2178 up-regulated genes and 850 down-regulated genes, respectively (Fig. 4a, Additional file 8: Table S8). The heatmap of the differentially expressed genes between IMPA and control is shown in Fig. 4b (Additional file 9: Table S9). Next, according to the conjoint analysis of RNA-seq and MeRIP-seq data, all genes were classified into 4 parts, including 24 up-regulated genes that were significantly hyper-methylated (10; up-hyper) or hypo-methylated (14; up-hypo) and 7 down-regulated genes that were significantly hyper-methylated (2; down-hyper) or hypo-methylated (5; down-hypo, |log_2_FC| > 1, and p < 0.05, Fig. 4c, Additional file 10: Table S10). These findings necessitate further exploration of the correlation of differentially methylated genes and differentially expressed gene levels in IMPA and normal samples. As illustrated in Fig. 4d (Additional file 11: Table S11), the genes were classed evenly into 10 groups according to the degree of expression, from low to high, the m^6^A density of each category was determined separately. An approximately positive correlation between m^6^A methylation and gene expression level before the 0.6 thresholds was determined, while the converse trend was observed for genes whose expression is over 0.6.

Furthermore, we also analyzed the expression of genes harboring differentially methylated m^6^A peaks in the five transcriptive regions between IMPA and control samples. In comparison with the control groups, the higher expression of mRNA specific to IMPA tissues in all five regions is also depicted in Fig. 4e (Additional file 12: Table S12). These results lay a solid groundwork for an in-depth research on m^6^A modifications in IMPA.

## Discussion

As an emerging field of post-transcriptional gene regulation, m^6^A modification has attracted extensive attention and interest in the research community. Studies have identified transcriptome-wide maps of m^6^A in multiple biological and pathological conditions, including maternal-to-zygotic transition, heart failure, and neurodegenerative disease [22–24]. It is noteworthy that m^6^A modification also plays an essential role in the development and drug responses of various cancers [25]. Through decades of research, global mapping of m^6^A in most kinds of cancers (e.g., hepatocellular carcinoma, glioblastoma, and breast cancer) has been achieved [25]. Nonetheless, to date, few studies on head and neck tumors have been conducted, and the distribution of m^6^A modification in IMPA at the transcriptome-wide level has not been fully elucidated.

In this study, we established an antibody-based methodology that presented transcriptome-wide m^6^A modification patterns in IMPA samples versus tumor-adjacent normal tissues, and we subsequently analyzed the alterations to gene expression and cancer-related pathways caused by aberrant m^6^A modifications. To the best of our knowledge, this report describes the first-ever high-throughput sequencing to comprehensively investigate RNA methylation in IMPA. Our data identified broad mRNA methylation and demethylation features over one of the most guarded prognosis subtypes, IMPA.

We observed abundant m^6^A methylation sites in the IMPA transcriptome by using the MeRIP-seq technique. The density was determined to be approximately 1.57 sites per transcript, which was significantly higher than that of sheep and pig, which exhibited approximately < 0.5 and < 0.6 m^6^A sites per transcript (Fig. 1c), respectively [26, 27]. Taken together, the m^6^A distribution density results imply that epigenetic modification may vary between different organisms or may be related to species differentiation. It is interesting to note that in all IMPA samples of this study, the m^6^A sites were also enriched around the CDS and in the vicinity of stop codons, close to the topological pattern of modification in colorectal cancer (Fig. 2b ii) [15]. As mentioned in the literature review, internal exon methylation would take effect on the alternative splicing. At the same time, translational regulation can be influenced by methylation near the stop codon [28]. Therefore, it can be assumed that m^6^A could play a vital role in controlling protein synthesis in IMPA. These findings, in combination with the results presented in Fig. 4d, offer some conceptual hypotheses in which m^6^A could regulate gene expression to a certain extent.

The most prominent finding to emerge from our data is that the difference in m^6^A modification between the tumor tissues and corresponding normal tissues was significant, which was also observed in breast cancer, non-small cell lung cancer, and pancreatic adenocarcinoma with different methylation levels [29–31]. This result suggests that the m^6^A modification could contribute to IMPA progression. A possible explanation for this global alteration of m^6^A modification patterns might be the unique expression of the key m^6^A regulators. Surprisingly and contrary to expectations, our current study did not detect significant differences among the key m^6^A regulators (writers, readers, and erasers) based on RNA-sEq. This result might be due to the relatively small sample size of our study or post-translational modifications [32]. Subsequent RNA-seq studies in IMPA are warranted to determine the actual reason.

RRACH and GGAC are proved to be over-represented motifs of m^6^A that are strongly preserved in humans [33–35]. Accordingly, in our current study, we identified multiple similar m^6^A consensus motifs in IMPA (Fig. 2d, e). These analyzed results were in keeping with the findings of a large number of previous studies in oral carcinomas, which suggested that we called specific m^6^A sites successfully [36].

As the most prevalent epigenetic modification in RNA, m^6^A has been proven to function as an essential mediator in tumorigenesis [37, 38]. Recently, Chen et al. reported that SOCS2 m^6^A methylation could contribute to its mRNA expression and liver carcinogenesis [39]. Additionally, the methylation of FOXMs serves to maintain the tumorigenicity of glioblastoma [40]. Indeed, in our study, we successfully identified a significant hyper-methylated characteristic in BACE2 (Table 2). Interestingly, in glioblastoma, patients with higher BACE2 expression exhibited an unfavorable outcome [41]. The GSEA database indicated that BACE2 could be involved in cell invasion and cell migration in gliomas [41]. Besides, compared with normal tissue, BACE2 was also observed to be over-expressed in breast and colon tumors [42, 43]. Furthermore, according to the post-transcriptional RNA modifications database, RMBase v2.0, BACE2 also shares an m^6^A methylation motif (Additional file 13: Fig. S1). Therefore, it may suggest that BACE2 affects IMPA progression via m^6^A modification manners. Further study is necessary to elucidate whether BACE2 methylation triggers the IMPA oncogenic process.

Prior studies have demonstrated that signaling pathways are significant targets to modulate tumor progression [44–46]. In our study, many critical biological pathways related to differentially methylated genes were enriched via GO, such as ATP transmembrane transporter activity, antiporter activity, and nucleic acid binding. Studies have revealed that these biological functions are closely implicated in the onset and development of tumors, including tumor growth, metastasis and chemo-resistance. For instance, high expression of ATP-binding cassette transmembrane transporters results in the efflux of cytotoxic agents from cancer cells, which leads to drug resistance. Qiu et al. demonstrated that the elevated expression of cellular nucleic acid-binding protein in fibrosarcoma cells down-regulated the transcription and expression of hnRNP K, which enhanced tumor cell death and suppressed tumor cell metastasis [47]. Based on KEGG analysis, genes with upregulated m^6^A modification were associated with mTOR signaling pathways in IMPA. It is intriguing to note that PI3K/AKT/mTOR pathways would contribute to cancer cell proliferation in head and neck squamous cell carcinoma (HNSCC), possibly serving a pro-tumorigenic function [48]. In accordance with the above HNSCC study, the most common malignant salivary gland neoplasm, adenoid cystic carcinoma, exhibits similar characteristics in the PI3K/AKT/mTOR axis [49]. One therapeutic method for tumors was to target the PI3K/AKT/mTOR pathways with an inhibitor [50]. Hence, it could be hypothesized that modulating the m^6^A modification of the transcripts of genes in the mTOR signaling pathways might provide a novel means of targeting IMPA for treatment.

Additionally, the carbohydrate and lipid metabolism have always been closely related to cancer cell proliferation and migration [51]. The KEGG pathway enrichment of coding genes harboring the hyper-methylated peaks in IMPA samples was related to the PPAR signaling pathways (Fig. 3c). The PPAR pathway may be involved in transcriptional regulation in many pathophysiological processes, which not only participate in fat and sugar metabolism, but also play roles in tumor development [52–55]. Moreover, researches have indicated a close association between the alteration of m^6^A modification and lipid metabolism, thereby, providing a link between m^6^A modification and IMPA tumorigenesis through metabolic ways [56–58]. Further studies, seeking to elucidate the potential mechanisms underlying PPAR signaling pathways in IMPA, are warranted.

In summary, we analyzed the differential m^6^A methylome in IMPA relative to the corresponding normal controls, and demonstrated a strong association between m^6^A modification and IMPA progression. Our study paves the way for future investigations aimed to explore the mechanism in the pathogenesis of IMPA.

## Supplementary Information

**Additional file 1. **Table S1. The differences and overlap of m^6^A modified genes in IMPA and corresponding normal tissues.

**Additional file 2.** Table S2. Number of peaks per transcript in IMPA and normal control tissues.

**Additional file 3.** Table S3. The estimation of hyper- and hypo-methylated peaks in IMPA samples compared with the control groups.

**Additional file 4.** Table S4. Number of altered m^6^A peaks in human chromosomes.

**Additional file 5.** Table S5. The enriched GO terms of the hyper-methylated genes.

**Additional file 6.** Table S6. The enriched GO terms of the hyper-methylated genes.

**Additional file 7.** Table S7. The enriched KEGG pathways of the hyper-methylated genes.

**Additional file 8.** Table S8. The distribution of genes with significant changes in mRNA levels.

**Additional file 9.** Table S9. The differentially expressed genes in IMPA and normal control tissues.

**Additional file 10.** Table S10. The distribution of genes with significant changes in both m^6^A modification and mRNA levels.

**Additional file 11.** Table S11. The estimation of m^6^A peaks density in IMPA.

**Additional file 12.** Table S12. Expression levels of genes containing m^6^A-specific peaks in different five regions of transcript between IMPA and normal tissues.

**Additional file 13: Fig. S1.** The RMBase v2.0 database shows the m^6^A modification motif in BACE2 based on GSE37003. motif sequence: GGACU.

## Data Availability

The datasets generated and/or analyzed during the current study are from the DAVID (https://david.ncifcrf.gov/) and RMBase v2.0 (http://rna.sysu.edu.cn/). MeRIP-seq and RNA-seq were uploaded to the GEO database (GSE161879), and can be accessed with the secure token key ejsdoiaujpehxyb.

## Abbreviations

PA: Pleomorphic adenoma
MPA: Malignant pleomorphic adenoma
IMPA: Invasive malignant pleomorphic adenoma
MeRIP-seq: Methylated RNA immunoprecipitation with next-generation sequencing
m^6^A: N6-Methyladenosine
RNA-seq: RNA sequencing
m^5^C: 5-Methylcytosine
m^1^A: N1-Methyladenosine
HNSCC: Head and neck squamous cell carcinoma
GO: Gene Oncology
KEGG: Kyoto Encyclopedia of Genes and Genomes
BP: Biological process
CC: Cellular component
MF: Molecular function
DAVID: The Database for Annotation Visualization and Integrated Discovery
IGV: Integrative Genomics Viewer
