# Supplementary figures and images for: Comprehensive analysis of the transcriptome‐wide m6A methylome in invasive malignant pleomorphic adenoma
Source: Cancer Cell Int. 2021 Mar 2;21:142. doi: 10.1186/s12935-021-01839-6 (PMC7923655; doi:10.1186/s12935-021-01839-6)

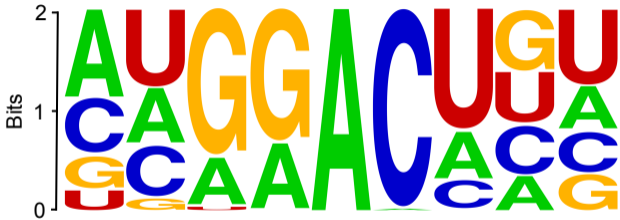

Supplement: Supplementary file 13 — Additional file 13: Fig. S1. The RMBase v2.0 database shows the m6A modification motif in BACE2 based on GSE37003. motif sequence: GGACU. [file 12935_2021_1839_MOESM13_ESM.pdf]
